# Supplementary figures and images for: A computational model to characterize the time-course of response to rapid antidepressant therapies
Source: PLoS One. 2024 Feb 2;19(2):e0297708. doi: 10.1371/journal.pone.0297708 (PMC10836665; doi:10.1371/journal.pone.0297708)

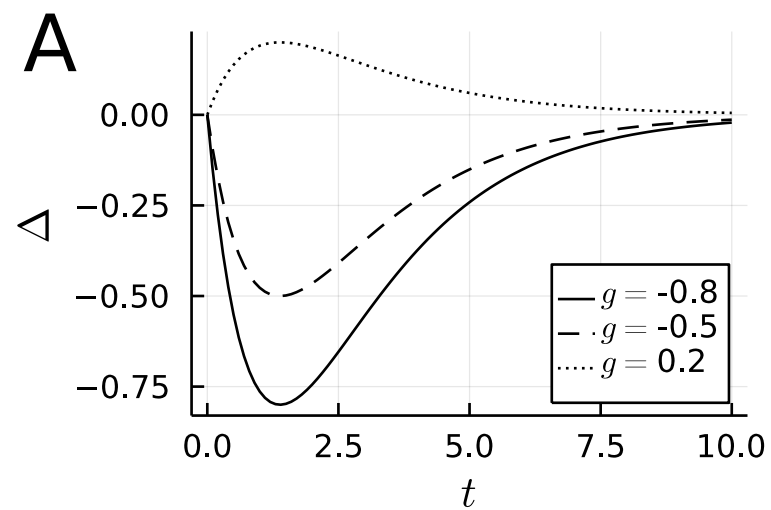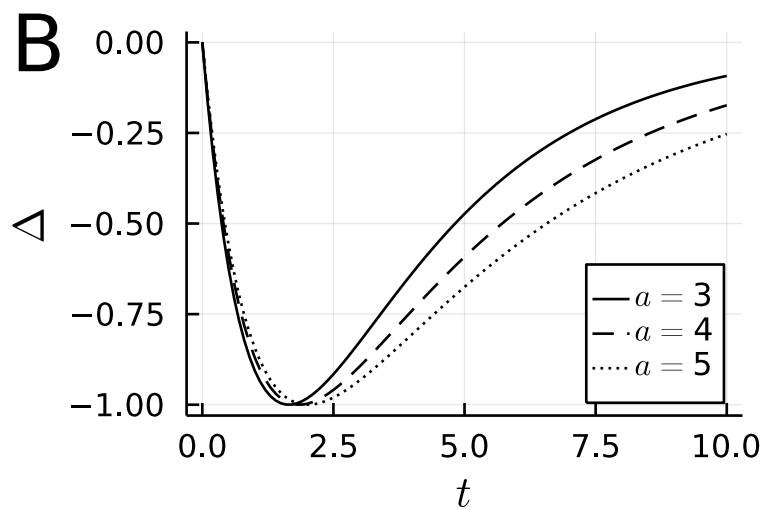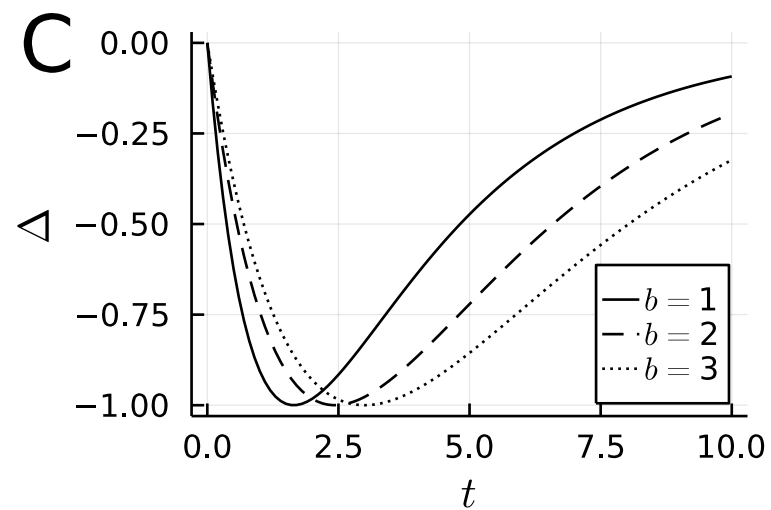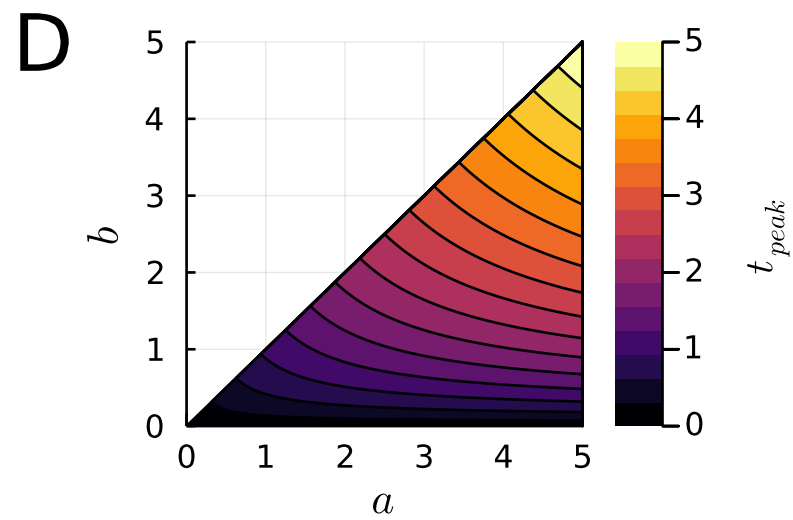

Supplement: S1 File — (ZIP) [file pone.0297708.s001.zip › rapid-antidepressant-timecourse-sim-main/Figure1.pdf]

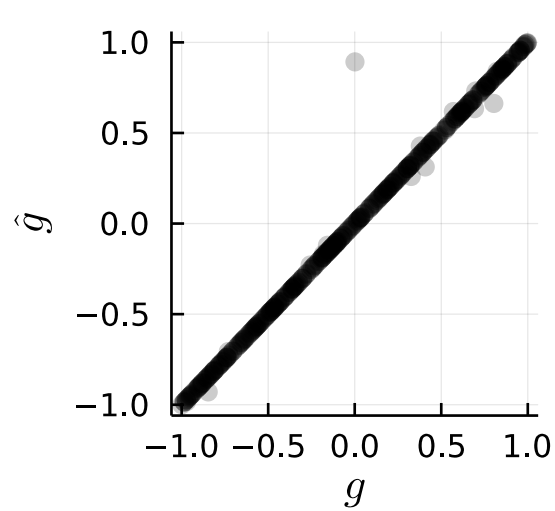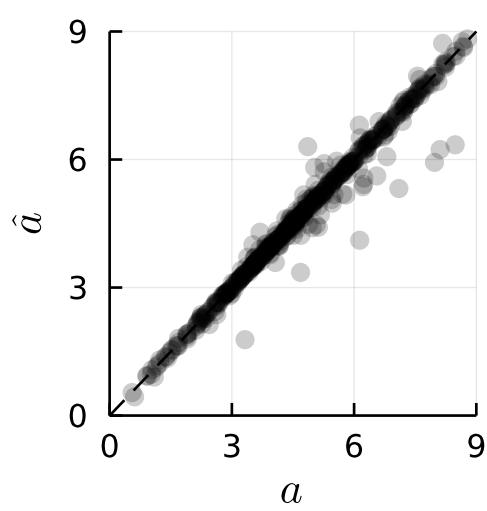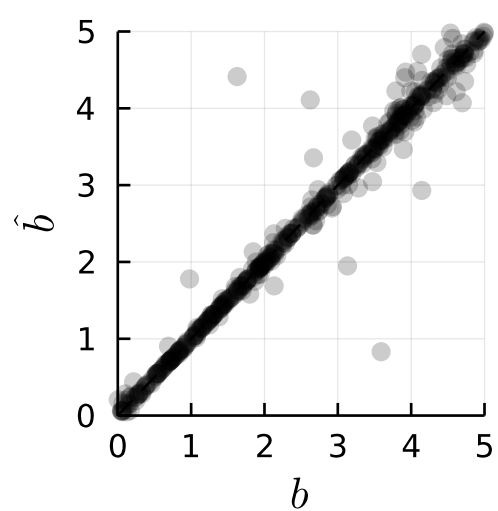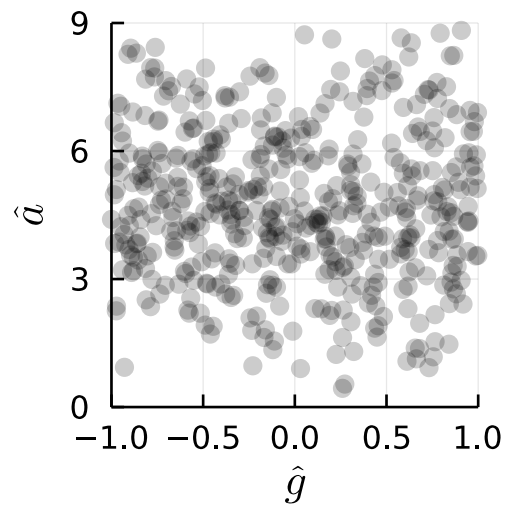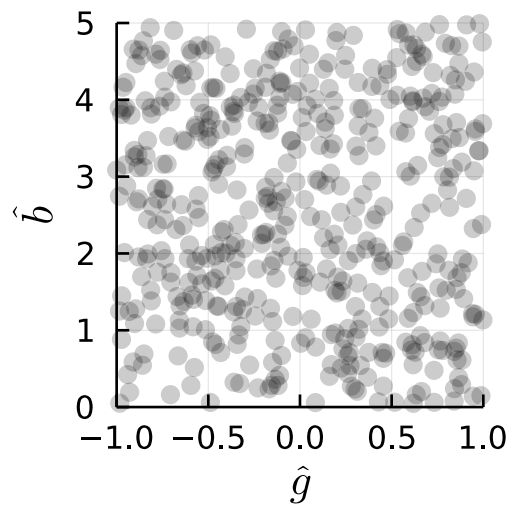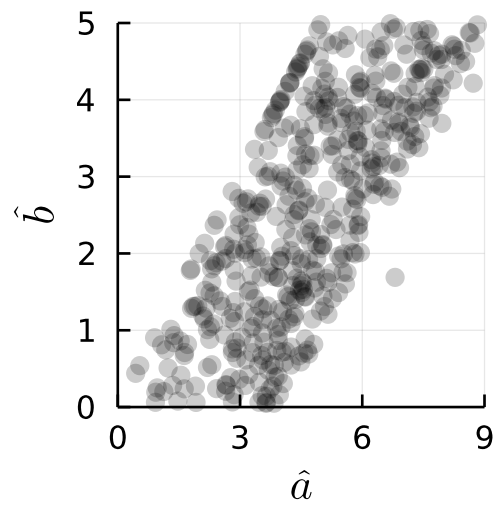

Supplement: S1 File — (ZIP) [file pone.0297708.s001.zip › rapid-antidepressant-timecourse-sim-main/Figure2.pdf]

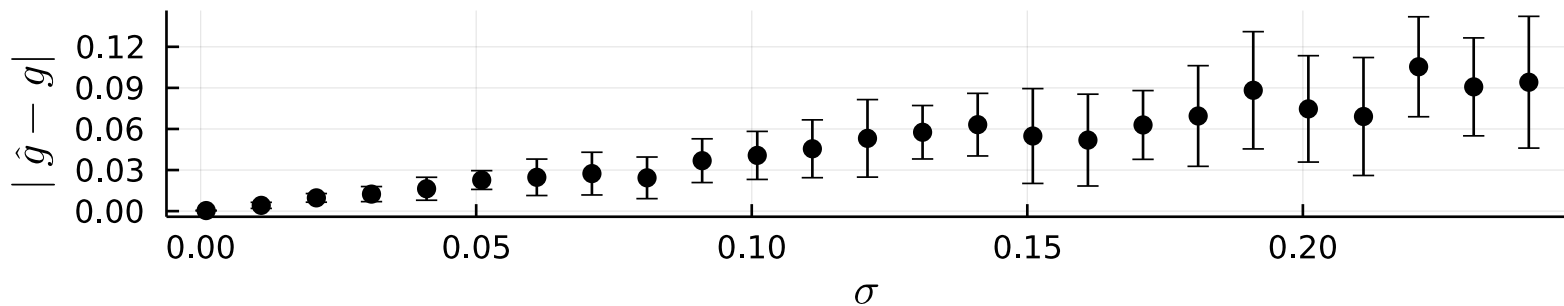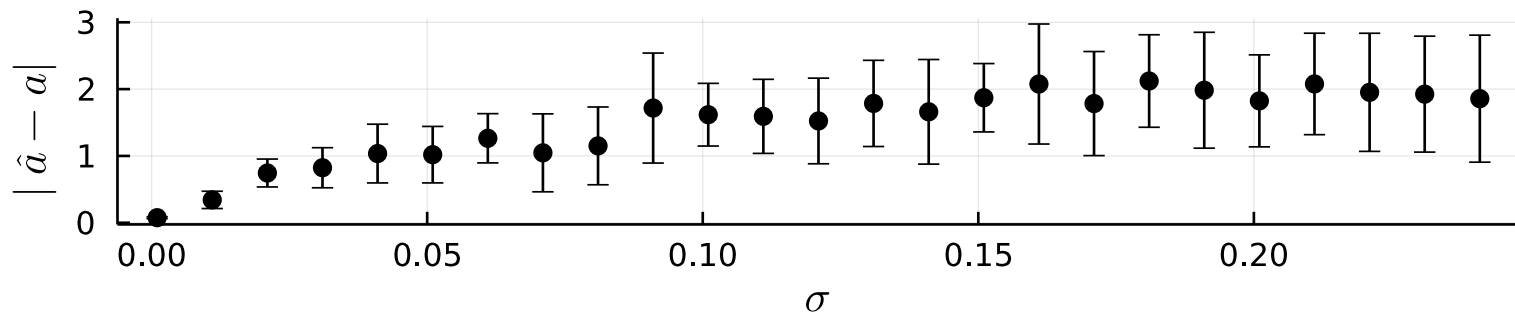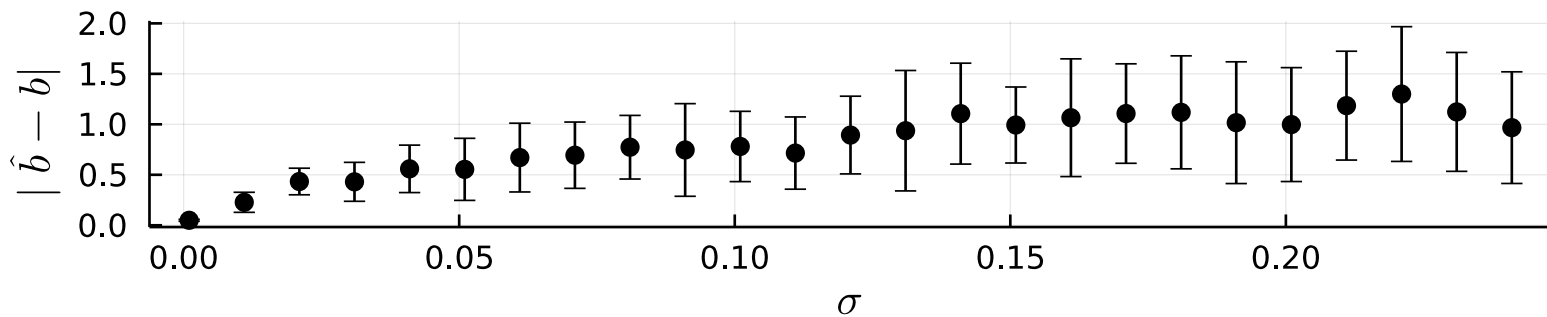

Supplement: S1 File — (ZIP) [file pone.0297708.s001.zip › rapid-antidepressant-timecourse-sim-main/Figure3.pdf]

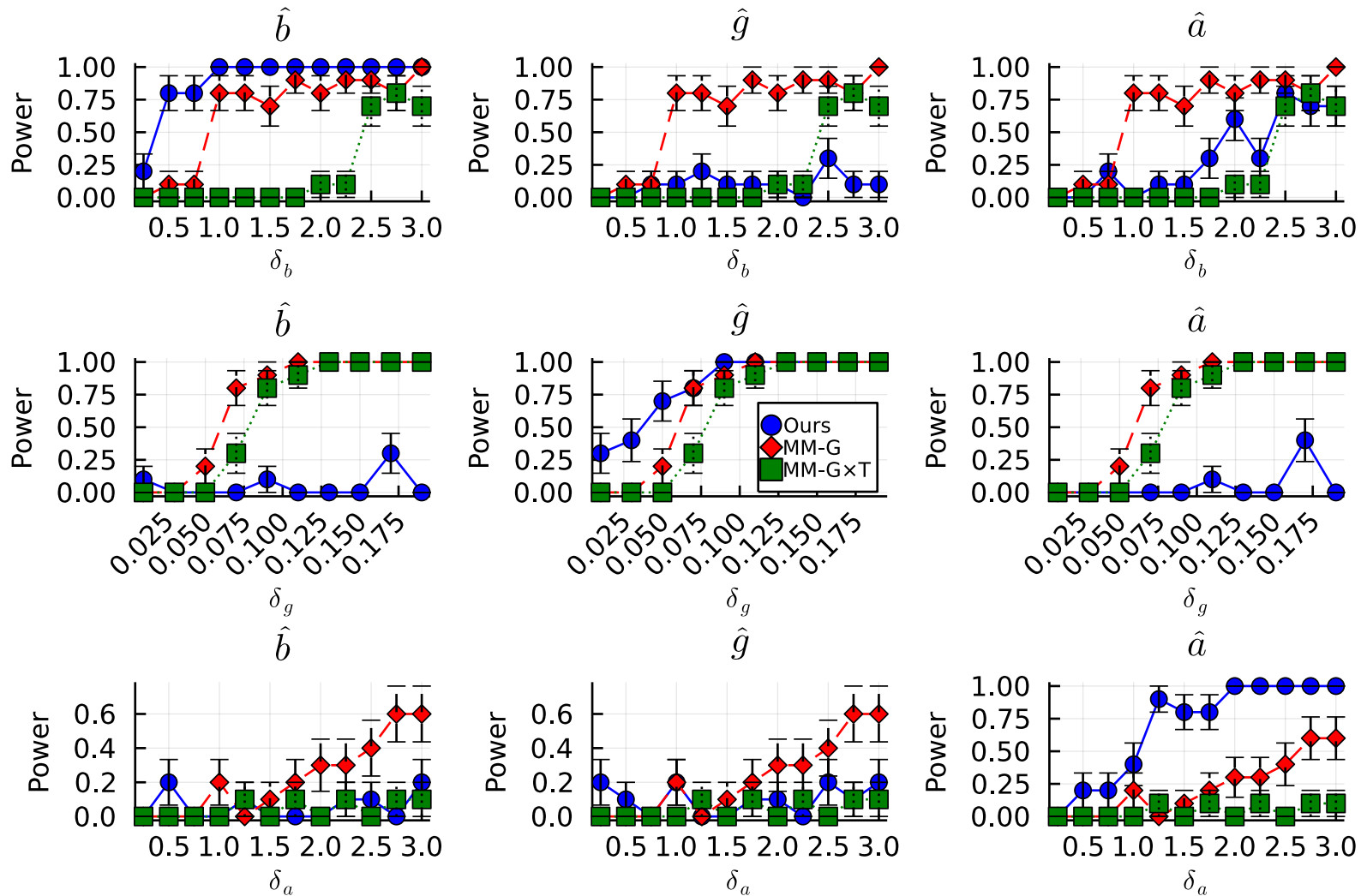

Supplement: S1 File — (ZIP) [file pone.0297708.s001.zip › rapid-antidepressant-timecourse-sim-main/Figure4.pdf]
