# Supplementary material for: A computational model to characterize the time-course of response to rapid antidepressant therapies
Source: PLoS One. 2024 Feb 2;19(2):e0297708. doi: 10.1371/journal.pone.0297708 (PMC10836665; doi:10.1371/journal.pone.0297708)
Supplement: Attachment — Submitted filename: ResponseToReviewers.pdf [file pone.0297708.s002.pdf]

A Computational Model to Characterize the Time-Course of Response to Rapid Antidepressant Therapies

Response to Reviewers

Abraham Nunes and Selena Singh

We thank the reviewers for their thoughtful and helpful responses. We have responded to each concern below, in order. We have also included excerpts of the changes incorporated into the manuscript along with each response.

Journal Requirements

Concern 1

Please ensure that your manuscript meets PLOS ONE's style requirements, including those for file naming. The PLOS ONE style templates can be found at https://journals.plos.org/plosone/s/file?id=wjVg/PLOSOne_formatting_sample_main_body.pdf and https://journals.plos.org/plosone/s/file?id=ba62/PLOSOne_formatting_sample_title_authors_affiliations.pdf

Thank you. We have updated the casing of the title. The word “Figure” has been changed to “Fig” throughout the paper.

Concern 2

Please note that PLOS ONE has specific guidelines on code sharing for submissions in which author-generated code underpins the findings in the manuscript. In these cases, all author-generated code must be made available without restrictions upon publication of the work. Please review our guidelines at https://journals.plos.org/plosone/s/materials-and-software-sharing#loc-sharing-code and ensure that your code is shared in a way that follows best practice and facilitates reproducibility and reuse.

Thank you. We have updated the data availability statement and included our repository as supplemental material.

Concern 3

We note that the grant information you provided in the ‘Funding Information’ and ‘Financial Disclosure’ sections do not match.

When you resubmit, please ensure that you provide the correct grant numbers for the awards you received for your study in the ‘Funding Information’ section.

4. Thank you for stating the following financial disclosure:
"Research Nova Scotia (AN); QEII Foundation (AN)"

Please state what role the funders took in the study. If the funders had no role, please state: "The funders had no role in study design, data collection and analysis, decision to publish, or preparation of the manuscript."
If this statement is not correct you must amend it as needed.
Please include this amended Role of Funder statement in your cover letter; we will change the online submission form on your behalf.

Thank you. We have included this in the cover letter. Please note that there was no funding number attached to the QEII Foundation grant. The Research Nova Scotia grant is RNS-NHIG-2021-1931. I have updated this in editorial manager but cannot find anywhere else to update it.

Concern 4

Please review your reference list to ensure that it is complete and correct. If you have cited papers that have been retracted, please include the rationale for doing so in the manuscript text, or remove these references and replace them with relevant current references. Any changes to the reference list should be mentioned in the rebuttal letter that accompanies your revised manuscript. If you need to cite a retracted article, indicate the article's retracted status in the References list and also include a citation and full reference for the retraction notice.

We have gone through the reference list. We do not note any papers that have been retracted. If you have information otherwise, please let us know and we will correct this.

Reviewer 1

Concern 1

Overall, the research paper you summarized appears to have made a significant attempt at developing a computational model that can enhance the understanding of responses to rapid antidepressant therapies.

The innovation of the computational model is a strong aspect of the paper. The claim of superiority rests on the paper's ability to demonstrate tangible advantages through the simulation results. This argument would be stronger with a broader comparison to multiple established models in the field, perhaps through a meta-analysis or systematic review, to give context to the model's performance relative to a wider spectrum of existing methodologies.

Thank you. There are currently no other models of which we are aware that are used in assessing the response to ketamine in clinical trials. Linear models are the standard. Conducting a systematic review and meta-analysis would be outside of the scope of the present study, but may be a worthwhile endeavour for future studies, once multiple analytical approaches are introduced.

Concern 2

The integration of computational modeling within the clinical context presents compelling utility. Nevertheless, the strength of this argument would be magnified by offering a roadmap outlining how the model's outcomes will translate into personalized medicine. For example, the paper might showcase how specific response profiles identified by the model can inform tailored treatment decisions, such as dosage adjustments or the choice of

therapeutic interventions. The paper seems to present a distinctly original approach through the design and implementation of this predictive model.

Thank you. We thought that we had presented somewhat of a roadmap in the discussion, but the reviewer has pointed out that it was not clear in the original form of the paper. We therefore updated the following paragraph in the discussion to bring more of a clear clinical context to the description.

> To our knowledge, none of the aforementioned predictors have been specifically associated with either magnitude, speed, or duration of response to ketamine. Yet, it is important to stratify predictors based on which features of the antidepressant response trajectory they predict. It is perhaps most important to find predictors of the duration/durability of rapid antidepressant response (which is encoded in our model using the parameter a). We must also seek to discover adjunctive or maintenance therapies for rapid antidepressants. Using our model-based approach, the effectiveness of these adjunctive or maintenance treatments can be measured directly based on their ability to prolong decay time a. Therefore, future studies should evaluate the degree to which biomarkers are specifically predictive of the magnitude (g), speed (b), and duration (a) of response to rapid antidepressant therapies. By doing so, we may better personalize the application of rapid antidepressant therapies to the needs of individual patients. For example, patients may demonstrate clinical features that predict rapid response (b) to one antidepressant, but sustained response (a) to another antidepressant. Understanding these predictive markers may facilitate devising optimal personalized combinations for such patients, with one agent used for rapid termination of the depressive episode while the other is used for sustaining response.
